# Cognitive theories of autism based on the interactions between brain functional networks

**DOI:** 10.3389/fnhum.2022.828985

**Published:** 2022-10-06

**Authors:** Sarah Barzegari Alamdari, Masoumeh Sadeghi Damavandi, Mojtaba Zarei, Reza Khosrowabadi

**Affiliations:** ^1^Institute for Cognitive and Brain Sciences, Shahid Beheshti University, Tehran, Iran; ^2^University of Southern Denmark, Neurology Unit, Odense, Denmark; ^3^Institute of Medical Science and Technology, Shahid Beheshti University, Tehran, Iran

**Keywords:** autism spectrum disorder, cognitive theories, resting-state fMRI, intrinsic connectivity networks, graph theory

## Abstract

Cognitive functions are directly related to interactions between the brain's functional networks. This functional organization changes in the autism spectrum disorder (ASD). However, the heterogeneous nature of autism brings inconsistency in the findings, and specific pattern of changes based on the cognitive theories of ASD still requires to be well-understood. In this study, we hypothesized that the theory of mind (ToM), and the weak central coherence theory must follow an alteration pattern in the network level of functional interactions. The main aim is to understand this pattern by evaluating interactions between all the brain functional networks. Moreover, the association between the significantly altered interactions and cognitive dysfunctions in autism is also investigated. We used resting-state fMRI data of 106 subjects (5–14 years, 46 ASD: five female, 60 HC: 18 female) to define the brain functional networks. Functional networks were calculated by applying four parcellation masks and their interactions were estimated using Pearson's correlation between pairs of them. Subsequently, for each mask, a graph was formed based on the connectome of interactions. Then, the local and global parameters of the graph were calculated. Finally, statistical analysis was performed using a two-sample *t*-test to highlight the significant differences between autistic and healthy control groups. Our corrected results show significant changes in the interaction of default mode, sensorimotor, visuospatial, visual, and language networks with other functional networks that can support the main cognitive theories of autism. We hope this finding sheds light on a better understanding of the neural underpinning of autism.

## Introduction

Autism spectrum disorder (ASD) is a neurodevelopmental disorder, which impacts social interactions and communication. It has a series of behavioral symptoms like restricted and repetitive behaviors, disability in making eye contact, and verbal disabilities. Several factors like genetics, biochemical, neuropsychological, and neurophysiological problems can cause ASD (Bosl et al., 2011; Ruggeri et al., 2014; West et al., 2014). ASD has an increasing prevalence with males 4.5 times as likely as their female counterparts (Christensen et al., 2016). ASD diagnosis is according to the criteria like DSM and ICD-R. The diagnosis is on the basis of a set of behavioral symptoms such as low social interaction including verbal and non-verbal communication, however, there is an ongoing debate on the ambiguity of the symptoms and their neural substrates (Lord et al., 1994; Ecker et al., 2013; Raff, 2014). New findings show that there is an association between ASD symptoms and functional connectivity networks of the brain (Kana et al., 2014).

The theory of mind (ToM) hypothesis and the weak central coherence theory are two cognitive theories that can explain behavioral and cognitive symptoms of autism. ToM explains the ability to have mental representations about one's own and other's inner emotional world. ToM hypothesis proposes that individuals with ASD are unable to understand the mental states of others (Baron-Cohen, 1988, 2004; Baron-Cohen et al., 1994). The weak central coherence theory of autism suggests that individuals with ASD are unable to “see the big picture.” They can focus on parts rather than the whole. Performance on visuospatial, perceptual, and verbal-semantic tasks, which need strong central coherence, is impaired in ASDs that support this theory (Ropar and Mitchell, 1999; Happe and Frith, 2006).

In recent decades, neuroimaging techniques are commonly used for observing brain activities. Modalities such as electroencephalography (EEG), functional magnetic resonance imaging (fMRI), and near-infrared spectroscopy (fNIRS) are non-invasive, reliable, and commonly used in ASD studies (Eldridge et al., 2014; Ichikawa et al., 2014; Wang et al., 2014; Devitt et al., 2015). Although it has a low temporal resolution, fMRI is preferred to EEG and fNIRS because of its high spatial resolution and also it can be performed in different mental states, task-based, or task-free (Smith et al., 2005; Zuo et al., 2010). Task-free or resting-state fMRI is used to evaluate different brain networks at the same time (Cole et al., 2010; Lee et al., 2013). To examine the changes in the intrinsic connectivity of different networks, resting-state fMRI is a convenient tool (Hull et al., 2017).

Functional connectivity networks extracted from resting-state fMRI data are called intrinsic connectivity networks (ICNs) and are highly related to cognitive processes (Laird et al., 2011). Some of the most common and widely indicated ICNs are primary, medial, and lateral visual networks, executive networks, salience network, right and left frontoparietal networks, sensorimotor network (SMN), auditory networks, and the default mode network (DMN); however, they change through studies (Smith et al., 2009; Bressler and Menon, 2010; Beckmann, 2012).

There are different methods for extracting intrinsic connectivity networks from resting-state fMRI data but one of the commonly used methods is independent component analysis (ICA; Supekar et al., 2008, 2010; Assaf et al., 2010; Bressler and Menon, 2010; Cole et al., 2010; Wiggins et al., 2011; Beckmann, 2012; Mueller et al., 2013; Starck et al., 2013; Di Martino et al., 2014; Dipasquale et al., 2015; Nomi and Uddin, 2015). The ICA approach can generally give the assumption of independence between the components (Beckmann, 2012; Lee et al., 2016). In this method, a mask (template) is applied to the data and a number of components are calculated through the correlation matrix (Wang et al., 2009; Liu et al., 2012; de Reus and van den Heuvel, 2013; Minati et al., 2013; Glasser et al., 2016). These components represent brain areas or networks. Different brain regions associated with a network are considered as a part of the same component. In ICA analysis, a single brain region can be found in multiple components. ICA is specifically useful for whole-brain connectivity analysis and despite ROI seed-based analysis is not limited to predefined brain networks (Beckmann and Smith, 2004; Cole et al., 2010; Hull et al., 2017). A cortical parcellation divides the cerebral cortex into subdivisions which are a set of areas that share special neurobiological properties. They thus provide basic maps for functional neuroimaging and many models of brain function are based on these abstractions. In parcellations, each region specifies a functionally distinct area which is of particular interest, as these encapsulate the fundamental neurobiological principles of functional specialization and segregation, and they form a basis for connectomics (Sporns et al., 2005).

Advances in resting-state analysis techniques have provided the possibility of examining the overall functional connectivity using graph theoretical methods. Brain connectivity networks can be assumed as graphs made up of nodes which can be regions or networks and edges that are functional connections between them (van den Heuvel et al., 2008; Bullmore and Sporns, 2009; Bressler and Menon, 2010; van den Heuvel and Hulshoff Pol, 2010; Sporns, 2011, 2013). Using these methods for resting-state fMRI, data consist of modeling cortical and sub-cortical areas as a set of nodes connected to each other by edges that represent the strength of the functional connectivity between node pairs. This information is expressed by an N × N matrix (connectivity matrix), where N is the number of nodes, and each element of the matrix shows the pairwise measure of connectivity. In analyzing ICNs data, the graph theoretical approach is one of the useful techniques (Sporns, 2011; Maximo et al., 2013). Since they can measure both global and local changes in network connectivity and strength, graph theory and network analysis techniques are applied in ASD studies (Wang et al., 2010; Hull et al., 2017).

Functional connectivity has been used vastly in ASD studies. These studies show changes in functional connectivity in ASD (Di Martino et al., 2008; Smith et al., 2013; von dem Hagen et al., 2013; Ray et al., 2014; Tyszka et al., 2014; Uddin et al., 2015; Olivito et al., 2016; Hull et al., 2017; Guo et al., 2019; Bathelt and Geurts, 2020). A brain region or network can be both under-connected with some areas and simultaneously over-connected with other areas in ASDs compared to healthy controls (HCs; Just et al., 2004; Cherkassky et al., 2006; Kennedy et al., 2006; Assaf et al., 2010; Jones et al., 2010; Weng et al., 2010; Di Martino et al., 2011; Delmonte et al., 2013; Keown et al., 2013; Redcay et al., 2013; Nebel et al., 2014; Cerliani et al., 2015; Chien et al., 2015; Holiga et al., 2019). There are under- and over-connectivity patterns associated with core ASD symptoms which are related to communication and daily living skills. These patterns are not affected by age, sex, or medication status. Under-connectivity is seen in sensory-motor regions and over-connectivity hubs are predominately in prefrontal and parietal cortices (Holiga et al., 2019). DMN, one of the resting-state networks, is active when the brain is in the resting state but deactivates during cognitive tasks or goal-directed behaviors (Raichle and Synder, 2007; Washington et al., 2014). According to different studies, under-connectivity and over-connectivity of DMN have been seen in ASD (Kennedy et al., 2006; Kennedy and Courchesne, 2008; Monk et al., 2009; Assaf et al., 2010; Weng et al., 2010; Wiggins et al., 2011; Rudie et al., 2013; Jung et al., 2014; Yerys et al., 2015; Lee et al., 2016; Olivito et al., 2016; Padmanabhan et al., 2017). DMN changes are more consistent findings in autistic individuals. Subregions of DMN are associated with cognitive functions such as mentalizing, memory, and understanding others' minds that are immediately relevant to cognitive theories of autism (Bathelt and Geurts, 2020). Studies have shown changes in the functional connectivity of other functional networks (Barttfeld et al., 2012; Rudie et al., 2013; Itahashi et al., 2014; Nebel et al., 2014; Verly et al., 2014; Uddin et al., 2015).

There are a number of studies that have used graph theory (Di Martino et al., 2013; Keown et al., 2013; Redcay et al., 2013; You et al., 2013; Ray et al., 2014; Balardin et al., 2015; Itahashi et al., 2015). A reduction in modularity, clustering, and local efficiency with increased local efficiency (shorter path lengths) has been detected (Rudie et al., 2013). An increase in local functional connections and betweenness centrality in the prefrontal region has been reported in adolescents with ASD (Redcay et al., 2013). The organization of the hub nodes has been changed in ASD (Itahashi et al., 2014; Balardin et al., 2015; Sadeghi et al., 2017). Changes in local and global parameters are contributed to the functional organization of the brain in individuals with ASD; however, they are inconsistent through studies.

On the basis of the international research literature on the changes in the brain's functional networks in ASD, the current study aimed to explore the interactions of whole-brain functional networks through four different parcellations. We evaluated the changes of the brain functional networks in ASDs compared to HCs.

Moreover, we explored how cognitive theories of autism can follow patterns of changes in the brain functional network. Prior studies just considered limited numbers of brain networks to investigate the functional interactions within or between the networks (Hull et al., 2017). In other words, this study was designed to discover (a) interactions of all brain functional networks (modular organization) in ASD subjects, as well as (b) the associations between the resting-state functional connectivity disorganizations of ASD with two of the main cognitive theories of autism, e.g., the theory of mind (ToM) and the weak central coherence theory.

## Materials and methods

### Participants

The included data in the current study were obtained from the ABIDE (http://fcon_1000.projects.nitrc.org/indi/abide/) database. We used the data sets contributed by the Georgetown University and New York University Langone Medical Center. One hundred six male and female subjects (5–14 years, 83 male and 23 female) were selected from both datasets and classified into two groups: (46 ASD, 41 male, and five female; 60 HC, 42 male, and 18 female).

ASD diagnosis included DSM-IV-TR criteria or DSM-V and confirmed with ADI-R1 and ADOS for the combined social and communication score. IQ (full, performance, and verbal IQ) scores were obtained for all participants using either the Wechsler Intelligence Scale for Children (WISC-IV) or Wechsler Abbreviated Scale of Intelligence (WASI) or the six subtests of the Differential Ability Scales, Second Edition (DAS-II; see Table 1). Table 1 presents statistics of Age, handedness, FIQ, PIQ, VIQ, ADOS (Autism Diagnostic Observation Schedule), and SRS (Social Responsiveness Scale). Gender and handedness are considered independent covariates.

**Table 1:** Demographics of the subjects.

**Index**	**ASD** **(mean ±sd/n)**	**HC** **(mean ±sd/n)**	**Statistics**	
			***p*-value**	***t*-value**
Age	(10.16 ± 2.68/46)	(10.04 ± 1.83/60)	0.785	0.274
Handedness	(1.23 ± 0.423/46)	(1.03 ± 0.181/60)	0.002	3.114
fIQ	(109.72 ± 16.36/46)	(118.98 ± 14.33/60)	0.002	3.101
vIQ	(112.54 ± 18.21/46)	(119.30 ± 16.05/60)	0.045	2.025
pIQ	(107.81± 16.49/46)	(114.48 ± 13.42/60)	0.032	2.177
SRS	(74.26 ± 16.00/46)	(44.26 ± 6.11/60)	0.000	13.320
ADOS	(10.23 ± 4.00/46)	0	0.000	19.852

sd, Standard deviation; vIQ, verbal IQ; pIQ, performance IQ; fIQ, full score IQ; ADOS-S, autism diagnostic observation schedule-social; SRS, social responsiveness scale.

For all participants, full-scale IQ below 80, other neurological diagnoses, use of antipsychotics, and contraindications for MRI or pregnancy were exclusionary. The original studies included in ABIDE received approval from each site's Institutional Review Board (IRB) and were performed aligned with the declaration of Helsinki protocols.

### Data acquisition

Participants of the New York University Langone Medical Center were scanned using a 3-Tesla Siemens Allegra following diagnostic assessment. During the resting-state fMRI scan, participants were asked to relax with their eyes open, while a white cross-hair against a black background was projected on a screen. Functional images were acquired in a T2^*^ MR sequence with the following parameters: TR/TE= 2,000/15 ms, 33 slices, 3.0 × 3.0 × 4.0 mm^3^ voxel size, 4 mm slice thickness, and the total volumes of 180 and flip angle= 90°. Anatomical images were acquired in a T1w MR sequence with the following parameters: TR/TE = 2,530/3.25 ms, 1.3 × 1.0 × 1.3 mm^3^ voxel size, and 1.33 mm slice thickness and flip angle = 7°.

Participants of the Georgetown University were scanned using a Siemens Trio 3-T scanner following diagnostic assessment. During the resting-state fMRI scan, participants were asked to relax with their eyes open, but to remain awake. Functional images were acquired in a T2^*^ MR sequence with the following parameters: TR/TE = 2000/30 ms, 43 slices, 3.0 × 3.0 × 2.5 mm^3^ voxel size, 2.5 mm slice thickness, flip angle = 90°, and the total volumes of 154. Anatomical images were acquired in a T1w MR sequence with the following parameters: TR/TE = 2,530/3.5 ms, 1.0 × 1.0 × 1.0 mm^3^ voxel size, 1.0 mm slice thickness, and flip angle = 7°.

### fMRI data preprocessing

We used the FMRIB software library (FSL: http://www.fmrib.ox.ac.uk/fsl; Jenkinson et al., 2012) and analysis of functional neuroimaging (AFNI: http://afni.nimh.nih.gov/afni) to perform standard preprocessing on imaging data (Cox, 1996). Structural images of all participants were deobliqued before reorientation to FSL-friendly space. Preprocessing steps for resting-state fMRI data were as follows: dropping the first five volumes, slice time correction for interleaved acquisitions (using Fourier interpolation), 3D motion correction (using least-squares alignment of three translational and three rotational parameters for 3D volume registration), despiking of extreme time series outliers (using a continuous transformation function), mean-based intensity normalization of all volumes by the same factor, spatial smoothing with Gaussian kernel of full-width half maximum 6 mm, temporal band-pass filtering between 0.009 and 0.01 Hz. Resting-state fMRI images were registered to structural images of the participants and then normalized to the MNI152 standard atlas using linear (FLIRT) and non-linear (FNIRT) transformations (9). Confounding time series of motion (three translational and three rotational parameters), white matter (WM), and cerebral spinal fluid (CSF) were then omitted from the data (Smith et al., 2013; Sadeghi et al., 2017; Lindquist et al., 2019).

We checked all structural MRI data, for motion artifacts visually. The resting-state fMRI data with movement of more than 4 mm were eliminated. From the two datasets, just 46 ASDs and 60 HCs could pass the quality control steps, mentioned above. Then the preprocessed fMRI data were used to construct the brain functional networks for each participant.

### Brain parcellation

In this study, we used the whole-brain parcellation method and laid four different masks (templates) on the fMRI data to compute a set number of brain functional networks, through the decomposition of the correlation matrix for each mask. Templates include 14 functionally parcellated networks (https://findlab.stanford.edu/functional_ROIs.html; Richiardi et al., 2015), seven functionally parcellated networks (Yeo et al., 2011), 17 functionally parcellated networks (Yeo et al., 2011), and 13 functionally parcellated networks (Thomason et al., 2011). The analysis presented in the main text body is based on the parcellation of Stanford University, while the results of other parcellations are presented in the Supplementary materials.

### Functional connectivity analysis

For each subject, the time series of each network were estimated by averaging the resting-state fMRI data of the nodes of a network. Then the functional interactions are measured by estimating temporal correlations between BOLD signals of every pair of the networks, defined by each template, and then a temporal correlation matrix was calculated using Pearson's correlation between the time series of every pair of networks. Then, an undirected weighted graph was calculated by considering each intrinsic connectivity network as a node and defining values of connectivity strength between the networks as the edges.

### Graph theoretical analyses

We used graph theoretical analysis for functional connectivity networks estimated in the previous step. Networks are supposed as the nodes of the graph and association between the networks, as the edges each functional network, also was characterized with local and global parameters. Local measures include: nodal degree, a measure of the number of connections (edges) of each node with the other network nodes; efficiency, which indicates the information transfer between nodes; local efficiency of an individual node is the inverse of the shortest path length connecting all the neighbors of that node; closeness centrality is calculated as the reciprocal of the sum of the length of the shortest paths between the node and all other nodes in the graph; betweenness centrality, which measures the number of shortest paths that pass through a node and indicates the importance of a node for efficient communication and integration across a network; and custering coefficient, which measures the rate of existing edges between the nearest neighbors vs. possible connections and reflects the presence of smaller subgraphs. Global measures include: clustering coefficient, the average path length, and a measure of global connectedness which indicates the average shortest connection length (i.e., the mean of the shortest path length values recorded for all the single node pairs); efficiency, global efficiency is the average of the local efficiency and includes all the nodes of the graph; and modularity, is the measure of the strength of divisions of a network into modules (Latora and Marchiori, 2001; Rubinove and Sporns, 2010; Sporns, 2014).

The brain connectivity toolbox (http://www.brain-connectivity-toolbox.net) was used for graph analysis (Rubinove and Sporns, 2010).

### Statistical analysis

**Figure 1:**
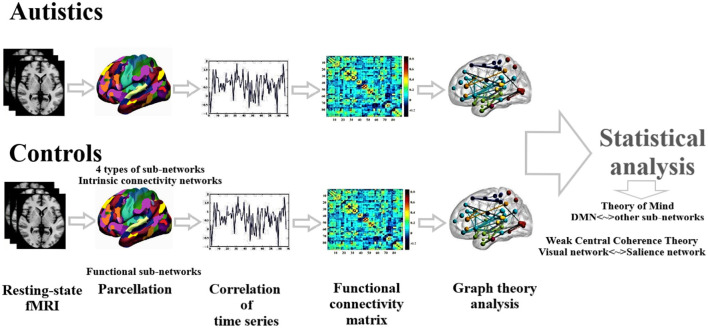
Schematic diagram of data analysis.

We applied a two-sample *t*-test on functional connectivity values and graph theoretical measures to compare ASD and HC statistically. Age, gender, handedness, and full IQ are considered independent covariates. The false discovery rate (FDR) method was applied to perform multiple comparisons correction (Storey and Tibshirani, 2003). Statistical analysis was carried out using SPSS software (IBM SPSS Statistics 22). Figure 1 presents the schematic diagram of data analysis.

## Results

As mentioned before, we used four different parcellations to extract functional networks and performed statistical comparisons separately for each parcellation. The comparison was carried out for functional connectivity values between pairs of networks and graph measures (local and global), between ASDs and HCs.

The results showed that there is no significant change in seven networks parcellation in ASDs compared to HCs but there are changes in functional connectivity values between some pairs of networks in 13, 14, and 17 networks parcellations. According to the results, in ASDs, there is a significant increase in functional connectivity between DMN and language networks and between primary-visual and posterior-salience networks but there is a decrease in functional connectivity between high-visual and sensorimotor networks and between visuospatial and salience networks (see Table 2). Table 2 presents the pairs of networks which are significantly different in ASDs compared to HCs, according to the parcellation used by Richiardi et al. (*p* < 0.05, FDR corrected).

**Table 2:** Significant changes in interactions between brain functional networks in ASD as compared to HC based on Richiardi et al. parcellation method.

**Networks**	**ASD** **(mean ±sd/n)**	**HC** **(mean ±sd/n)**	**Effect size**	***p*-value**	***t*-value**	**Observed power**
Post-salience and Visuospatial	(0.3390 ± 0.2440/46)	(0.3466 ± 0.2238/60)	0.028	0.046	2.020	0.516
High-visual and Sensorimotor	(0.4366 ± 0.2042/46)	(0.5246 ± 0.1632/60)	0.431	0.014*	2.499	0.697
Language and Ventral-DMN	(0.4543 ± 0.1551/46)	(0.3711 ± 0.1926/60)	0.535	0.001*	3.380	0.917
Post-salience and Prim-visual	(0.3807 ± 0.2486/46)	90.3404 ± 0.2438/60)	0.161	0.012*	2.548	0.713

sd, standard deviation.

*Denotes P-value < 0.05, FDR corrected.

In global graph measures, there is no significant change between the two groups. In local graph measures, the results indicate the decrement of betweenness centrality in visuospatial, high visual, and networks in ASDs compared to HCs through four parcellations (*p* < 0.05, FDR corrected). Table 3 presents the networks that have a significant decrease in betweenness centrality in ASDs compared to HCs, according to the parcellation used by Richiardi et al. (*p* < 0.05, FDR corrected). The results of the parcellations used by Yeo et al. and Thomason et al. are presented in the Supplementary materials.

**Table 3:** Significant decreases in betweenness centrality of brain networks in ASD as compared to HC based on Richiardi et al. parcellation method.

**Networks**	**ASD** **(mean ±sd)**	**HC** **(mean ±sd)**	**Effect size**	***p*-value**	***t*-value**	**Observed power**
Visuospatial	4.610 ± 6.238	7.300 ± 8.083	0.431	0.019*	2.381	0.655
High-visual	3.870 ± 5.833	4.530 ± 6.878	0.113	0.040	2.077	0.539

sd, standard deviation.

*Denotes P-value < 0.05, FDR corrected.

**Figure 2:**
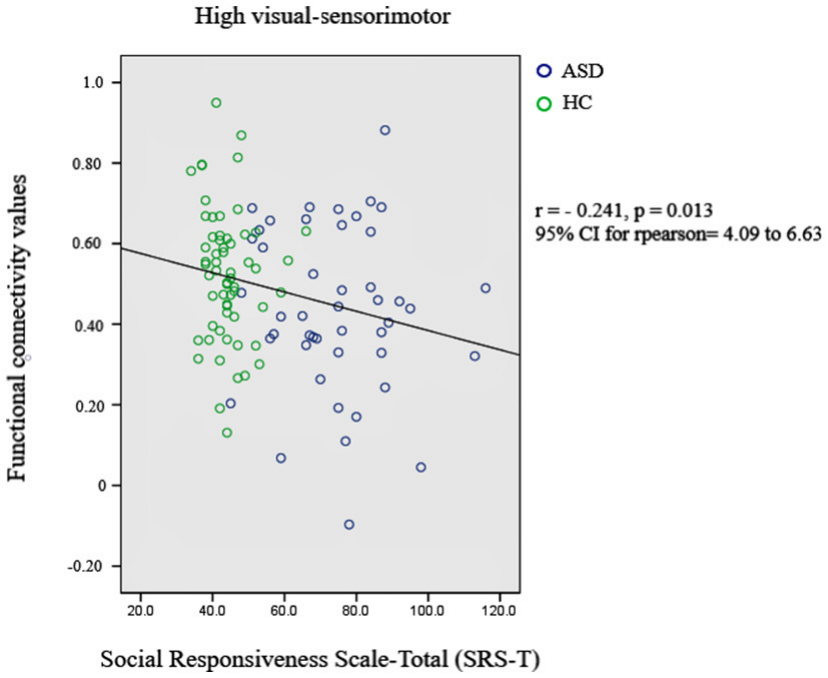
Relationship between significant changes in functional connectivity between high-visual and sensorimotor networks and autism diagnosis criteria.

Subsequently, the relationship between significant changes in functional connectivity between the functional networks and the social responsiveness scale (SRS) was also checked. A score of 76 or higher indicates deficiencies in reciprocal social behavior that are clinically significant. Such scores are strongly associated with the clinical diagnosis of autism spectrum disorder. The significant decrement in the functional connectivity between high-visual and sensorimotor networks is correlated with the SRS-total scores of autistic subjects. Figure 2 presents the most significant result.

## Discussion

In this study, we used resting-state fMRI data to define brain functional networks. We calculated functional connectivity between pairs of networks and graph theoretical measures (global and local) to study interactions between them in ASDs compared with HCs. According to the results, there are alterations in functional connectivity between some pairs of networks. DMN, language, visual, visuospatial, posterior-salience, frontoparietal, and sensorimotor networks have the most alterations. Although the anatomical properties of these networks are not exactly the same through different parcellations, they are more or less comparable. Based on the results, there is a significant increase in functional connectivity between DMN and language, posterior-salience, and frontoparietal networks in ASDs. These results are consistent with previous studies (Kennedy and Courchesne, 2008; Redcay et al., 2013; Rudie and Dapretto, 2013). Functional connectivity between visual network and sensorimotor network and between posterior-salience and visuospatial networks has significantly decreased, but there is an increase in functional connectivity between posterior-salience and visual networks, which have been reported in previous studies (Ebisch et al., 2011; Cerliani et al., 2015; Lee et al., 2016; Sadeghi et al., 2017). In addition, the results indicate the decrease of betweenness centrality in somatomotor, IFG-middle-temporal, visuospatial, high-visual, dorsal attention, and limbic networks in ASDs compared with HCs, which have also been reported (Cherkassky et al., 2006; Gotts et al., 2012; Rudie et al., 2013; Sadeghi et al., 2017).

Although functional connectivity between visual networks and some mentioned networks increases but the decrement of betweenness centrality of this network shows the decrement of its total interactions. In addition, language, visual, and attention networks that have increased functional connectivity with DMN show decreased betweenness centrality which indicates a relationship between the increase of functional connectivity with DMN and the decrease in total interactions. Such a finding has been reported in other studies (Verly et al., 2014; Yerys et al., 2015).

As mentioned above, DMN is involved in attention to internal emotional states, self-referential processing and highly responds to the theory of mind (ToM) and social cognition (Bressler and Menon, 2010; Laird et al., 2011; Yeo et al., 2014; Bathelt and Geurts, 2020) which are notably impaired in ASDs. The changes in the functional connectivity of DMN with other networks can be associated with the symptoms like inability to understand others, ToM, perception of social behavior, and hyper-sensitivity (Hull et al., 2017; Holiga et al., 2019); Unlike DMN, salience network is involved in survival-relevant events in the environment (Bressler and Menon, 2010; Laird et al., 2011; Yeo et al., 2014) and alterations of this network can be the cause of impaired attention in ASDs. Alterations in interactions between language network (IFG-middle-temporal) and limbic network in ASDs can be associated with impairments in social behavior and interactions and language, which has also been reported in other studies (Gotts et al., 2012; Yerys et al., 2015). In addition, the limbic network is involved in the discrimination of emotional faces and emotional pictures especially those related to fear, cheer, and humor (Laird et al., 2011) which is notably impaired in ASDs.

As mentioned above, according to The weak central coherence, ASDs can focus on parts rather than the whole, which means they are not able to “see the big picture” and concentrate on the whole (Happe and Frith, 1996; Ropar and Mitchell, 1999). The alterations of total interaction between visuospatial and high-visual networks and also the changes in interactions between posterior-salience and visuospatial, in ASDs can support this theory (Ropar and Mitchell, 1999; Happe and Frith, 2006; Laird et al., 2011). Visual, visuospatial, and sensorimotor networks are the networks that have a mixture of functions related to stimuli integration coordination and execution. Their interactions are related to cognitive control of visuomotor timing and preparation of executed movements and are highly related to behavioral domains such as action imagination, perception, and visual motion. They respond to stimuli such as visual targets and fixation points (Laird et al., 2011). Thus, the changes in the interactions of these networks in ASDs can be related to the repetitive and aimless movement behavior in ASDs. In this study, we explored the relationship between the resting-state functional connectivity disorganizations of ASD with cognitive theories of ASD based on the previous studies in the literature. We just suggest that functional disorganizations of ASD are related to cognitive theories of ASD and subsequently to the quantitative scores of behavioral representations. Of course, extensive future studies are needed to test these hypotheses by studying the relationships between such functional disorganizations and the quantitative scores of behavioral representations. Moreover, careful consideration of the sample size of the participants is also suggested.

## Conclusion

We analyzed functional connectivity values and global and local parameters of the brain connectome in ASDs and HCs and compared them with each other. According to the analyses of the functional connectivity between pairs of networks, there is a significant increase in functional connectivity between DMN and language networks, and visual and ventral attention networks. Moreover, functional connectivity between visual networks and frontoparietal and salience networks increases but there is a decrease in functional connectivity between visual and sensorimotor networks. Although the global functional connectivity measures have alterations in ASDs compared to HCs, these changes are not significant. In local measures, there is a decrease in betweenness centrality of sensorimotor, visuospatial, visual, dorsal attention, language, and limbic networks, through four parcellations. In summary, there are alterations of interactions between brain functional connectivity networks in ASDs. These alterations are different due to the heterogeneous nature of autism and age, gender, and IQ of the participants and analysis methods. DMN, sensorimotor, visuospatial, visual, and language networks have the most alteration. These changes can be associated with the cognitive and behavioral impacts of autism which are explained by cognitive theories, such as the theory of mind and the weak central coherence.

## Data availability statement

The original contributions presented in the study are included in the article/Supplementary material, further inquiries can be directed to the corresponding author.

## Ethics statement

The studies involving human participants were reviewed and approved by Georgetown University New York University Langone Medical Center. Written informed consent to participate in this study was provided by the participants' legal guardian/next of kin.

## Author contributions

SA and MS were responsible for data gathering, processing, and article writing, while RK and MZ was responsible for experimental design, article modification, and proofreading. All authors contributed to the article and approved the submitted version.

## Conflict of interest

The authors declare that the research was conducted in the absence of any commercial or financial relationships that could be construed as a potential conflict of interest.

## Publisher's note

All claims expressed in this article are solely those of the authors and do not necessarily represent those of their affiliated organizations, or those of the publisher, the editors and the reviewers. Any product that may be evaluated in this article, or claim that may be made by its manufacturer, is not guaranteed or endorsed by the publisher.

## References

[B1] Assaf M. Jagannathan K. Calhoun V. D. Miller L. Stevens M. C. Sahl R.. (2010). Abnormal functional connectivity of default mode subnetworks in autism spectrum disorder patients. Neuroimage 53, 247–256. 10.1016/j.neuroimage.2010.05.067 20621638 PMC3058935

[B2] Balardin J. B. Comfort W. E. Daly E. Murphy C. Andrews D. Murphy D. G. M.. (2015). Decreased centrality of cortical volume covariance networks in autism spectrum disorders. J. Psychiatr. Res. 69, 142–149. 10.1016/j.jpsychires.2015.08.003 26343606

[B3] Baron-Cohen S. (1988). Without a theory of mind one cannot participate in a con-versation. Cognition 29, 83–84. 10.1016/0010-0277(88)90011-X 3402194

[B4] Baron-Cohen S. (2004). The cognitive neuroscience of autism. J. Neurol. Neurosurg. Psychiatr. 75, 945–948. 10.1136/jnnp.2003.018713 15201345 PMC1739089

[B5] Baron-Cohen S. Ring H. Moriarty J. Schmitz B. Costa D. Ell P.. (1994). Recognition of mental state terms – clinical findings in children with autism and a functional neuroimaging study of normal adults. Br. J. Psychiatr. 165, 640–649. 10.1192/bjp.165.5.640 7866679

[B6] Barttfeld P. Wicker B. Cukier S. Navarta S. Lew S. Leiguarda R.. (2012). State-dependent changes of connectivity patterns and functional brain network topology in autism spectrum disorder. Neuropsychologia 50, 3653–3662. 10.1016/j.neuropsychologia.2012.09.047 23044278

[B7] Bathelt J. Geurts H. M. (2020). Difference in default mode network subsystems in autism across childhood and adolescence. Autism 556–565. 10.1177/1362361320969258 33246376 PMC7874372

[B8] Beckmann C. F. (2012). Modelling with independent components. Neuroimage 62, 891–901. 10.1016/j.neuroimage.2012.02.020 22369997

[B9] Beckmann C. F. Smith S. M. (2004). Probabilistic independent component analysis for functional magnetic resonance imaging. IEEE Trans. Med. Imaging 23, 137–152. 10.1109/TMI.2003.822821 14964560

[B10] Bosl W. Tierney A. Tager-Flusberg H. Nelson C. E. E. G. (2011). complexity as a biomarker for autism spectrum disorder risk. BMC Med. 9, 18. 10.1186/1741-7015-9-18 21342500 PMC3050760

[B11] Bressler S. L. Menon V. (2010). Large scale brain networks in cognition: emerging methods and principles. Trends Cogn. Sci. 14, 277–292. 10.1016/j.tics.2010.04.004 20493761

[B12] Bullmore E. Sporns O. (2009). Complex brain networks: graph theoretical analysis of structural and functional systems. Nat. Rev. Neurosci. 10, 186–198. 10.1038/nrn2575 19190637

[B13] Cerliani L. Mennes M. Thomas R. M. Di Martino A. Thioux M. Keysers C.. (2015). Increased functional connectivity between subcortical and cortical rest-ing-state networks in autism spectrum disorder. J. Am. Med. Assoc. Psychiatr. 72, 767–777. 10.1001/jamapsychiatry.2015.0101 26061743 PMC5008437

[B14] Cherkassky V. L. Kana R. K. Keller T. A. Just M. A. (2006). Functional connectivity in a baseline resting-state network in autism. Neuroreport 17, 1687–1690. 10.1097/01.wnr.0000239956.45448.4c 17047454

[B15] Chien H. Y. Lin H. Y. Lai M. C. Gau S. S. Tseng W. Y. (2015). Hyperconnectivity of the right posterior temporo-parietal junction predicts social difficulties in boys with autism spectrum disorder. Autism Res. 8, 427–441. 10.1002/aur.1457 25630517

[B16] Christensen D. L. Bilder D. A. Zahorodny W. Pettygrove S. Durkin M. S. Fitzgerald R. T.. (2016). Prevalence and characteristics of autism spectrum disorder among children aged 8 years – autism and developmental disabilities monitoring network. MMWR Surveill. Summ. 65, 1–23. 10.15585/mmwr.ss6503a1 27031587 PMC7909709

[B17] Cole D. M. Smith S. M. Beckmann C. F. (2010). Advances and pitfalls in the analysis and interpretation of resting-state FMRI data. Front. Syst. Neurosci. 4, 8. 10.3389/fnsys.2010.00008 20407579 PMC2854531

[B18] Cox R. (1996). WAFNI software for analysis and visualization of functional magnetic resonance neuroimages. Comput. Biomed. Res. 29, 162–173. 10.1006/cbmr.1996.0014 8812068

[B19] de Reus M. A. van den Heuvel M. P. (2013). The parcellation-based connectome: limitations and extensions. Neuroimage 80, 397–404. 10.1016/j.neuroimage.2013.03.053 23558097

[B20] Delmonte S. Gallagher L. O'Hanlon E. McGrath J. Balsters J. H. (2013). Functional and structural connectivity of frontostriatal circuitry in autism spectrum disorder. Front. Hum. Neurosci. 7, 430. 10.3389/fnhum.2013.00430 23964221 PMC3734372

[B21] Devitt N. M. Gallagher L. Reilly R. B. (2015). Autism spectrum disorder (ASD) and fragile X syndrome (FXS): two overlapping disorders reviewed through electroencephalography-what can be interpreted from the available information? Brain Sci. 5, 92–117. 10.3390/brainsci5020092 25826237 PMC4493458

[B22] Di Martino A. Kelly C. Grzadzinski R. Zuo X. N. Mennes M. Mairena M. A.. (2011). Aberrant striatal functional connectivity in children with autism. Biol. Psychiatr. 69, 847–856. 10.1016/j.biopsych.2010.10.029 21195388 PMC3091619

[B23] Di Martino A. Scheres A. Margulies D. S. (2008). Functional connectivity of human striatum: a resting state FMRI study. Cereb. Cortex 18, 2735–2747. 10.1093/cercor/bhn041 18400794

[B24] Di Martino A. Yan C. G. Li Q. Denio E. Castellanos F. X. Alaerts K.. (2014). The autism brain imaging data exchange: towards a large-scale evaluation of the intrinsic brain architecture in autism. Mol. Psychiatr. 19, 659–667. 10.1038/mp.2013.78 23774715 PMC4162310

[B25] Di Martino A. Zuo X. N. Kelly C. Grzadzinski R. Mennes M. Schvarcz A.. (2013). Shared and distinct intrinsic functional network centrality in autism and attention-deficit/hyperactivity disorder. Biol. Psychiatr. 74, 623–632. 10.1016/j.biopsych.2013.02.011 23541632 PMC4508007

[B26] Dipasquale O. Griffanti L. Clerici M. (2015). High-dimensional ICA analysis detects within-network functional connectivity damage of default-mode and sensory-motor networks in Alzheimer's disease. Front. Hum. Neurosci. 9, 43. 10.3389/fnhum.2015.00043 25691865 PMC4315015

[B27] Ebisch S. J. Gallese V. Willems R. M. Mantini D. Groen W. B. Romani G. L.. (2011). Altered intrinsic functional connectivity of anterior and posterior insula regions in high-functioning participants with autism spectrum disorder. Hum. Brain Mapp. 32, 1013–1028. 10.1002/hbm.21085 20645311 PMC6870194

[B28] Ecker C. Spooren W. Murphy D. G. M. (2013). Translational approaches to the biology of Autism: false dawn or a new era[quest]. Mol. Psychiatr. 18, 435–442. 10.1038/mp.2012.102 22801412 PMC3606942

[B29] Eldridge J. Lane A. E. Belkin M. Dennis S. (2014). Robust features for the automatic identification of autism spectrum disorder in children. J. Neurodevelopment. Disord. 6, 12. 10.1186/1866-1955-6-12 24936212 PMC4039057

[B30] Glasser M. F. Coalson T. S. Robinson E. C. (2016). A multi-modal parcellation of human cerebral cortex. Nature 536, 171–178. 10.1038/nature18933 27437579 PMC4990127

[B31] Gotts S. J. Simmons W. K. Milbury L. A. Wallace G. L. Cox R. W. Martin A.. (2012). Fractionation of social brain circuits in autism spectrum disorders. Brain 135, 2711–2725. 10.1093/brain/aws160 22791801 PMC3437021

[B32] Guo X. Duan X. Heng A. Chen H. He C. Xiao J.. (2019). Altered inter- and intrahemispheric functional connectivity dynamics in autistic children. Hum. Brain Mapp. 2019, 1–10. 10.1002/hbm.24812 31600014 PMC7268059

[B33] Happe F. Frith U. (1996). The neuropsychology of autism. Brain. 119, 1377–1400. 8813299 10.1093/brain/119.4.1377

[B34] Happe F. Frith U. (2006). The weak coherence account: detail-focused cognitive style in autism spectrum disorders. J. Autism Dev. Disord. 36, 5–25. 10.1007/s10803-005-0039-0 16450045

[B35] Holiga S. Hipp J. F. Chatham C. H. Garces P. Spooren W. Liogier D'Ardhuy X.. (2019). Patients with autism spectrum disorders display reproducible functional connectivity alterations. Sci. Transl. Med. 11, 9223. 10.1126/scitranslmed.aat9223 30814340

[B36] Hull J. V. Jacokes Z. J. Torgerson C. M. Irimia A. Van Horn J. D. (2017). Resting-state functional connectivity in autism spectrum disorders: a review. Front. Psychiatr. 7, 205. 10.3389/fpsyt.2016.00205 28101064 PMC5209637

[B37] Ichikawa H. Kitazono J. Nagata K. Manda A. Shimamura K. Sakuta R.. (2014). Novel method to classify hemodynamic response obtained using multi-channel fNIRS measurements into two groups: exploring the combinations of channels. Front. Hum. Neurosci. 8, 480. 10.3389/fnhum.2014.00480 25071510 PMC4078995

[B38] Itahashi T. Yamada T. Watanabe H. Nakamura M. Jimbo D. Shioda S.. (2014). Altered network topologies and hub organization in adults with autism: a resting-state fMRI study. PLoS ONE 9, e94115. 10.1371/journal.pone.0094115 24714805 PMC3979738

[B39] Itahashi T. Yamada T. Watanabe H. Nakamura M. Ohta H. Kanai C.. (2015). Alterations of local spontaneous brain activity and connectivity in adults with high-functioning autism spectrum disorder. Mol. Autism 6, 30. 10.1186/s13229-015-0026-z 26023326 PMC4446946

[B40] Jenkinson M. Beckmann C. F. Behrens T. E. Woolrich M. W. Smith S. M. (2012). Fsl. Neuroimage. 62, 782–790. 10.1016/j.neuroimage.2011.09.015 21979382

[B41] Jones T. B. Bandettini P. A. Kenworthy L. Case L. K. Milleville S. C. Martin A.. (2010). Sources of group differences in functional connectivity: an investigation applied to autism spectrum disorder. Neuroimage 49, 401–414. 10.1016/j.neuroimage.2009.07.051 19646533 PMC2832835

[B42] Jung M. Kosaka H. Saito D. N. Ishitobi M. Morita T. Inohara K.. (2014). Default mode network in young male adults with autism spectrum disorder: relationship with autism spectrum traits. Mol. Autism 5, 35. 10.1186/2040-2392-5-35 24955232 PMC4064274

[B43] Just M. A. Cherkassky V. L. Keller T. A. Minshew N. J. (2004). Cortical activation and synchronization during sentence comprehension in high-functioning autism: evidence of underconnectivity. Brain 127, 1811–1821. 10.1093/brain/awh199 15215213

[B44] Kana R. K. Uddin L. Q. Kenet T. Chugani D. Muller R. A. (2014). Brain connectivity in autism. Front. Hum. Neurosci. 8, 349. 10.3389/fnhum.2014.00349 24917800 PMC4041005

[B45] Kennedy D. P. Courchesne E. (2008). The intrinsic functional organization of the brain is altered in autism. Neuroimage 39, 1877–1885. 10.1016/j.neuroimage.2007.10.052 18083565

[B46] Kennedy D. P. Redcay E. Courchesne E. (2006). Failing to deactivate: resting functional abnormalities in autism. Proc. Natl. Acad. Sci. U. S. A. 103, 8275–8280. 10.1073/pnas.0600674103 16702548 PMC1472462

[B47] Keown C. L. Shih P. Nair A. Peterson N. Mulvey M. E. Müller R. A.. (2013). Local functional overconnectivity in posterior brain regions is associated with symptom severity in autism spectrum disorders. Cell Rep. 5, 567–572. 10.1016/j.celrep.2013.10.003 24210815 PMC5708538

[B48] Laird A. R. Fox P. M. Eickhoff S. B. Turner J. A. Ray K. L. McKay D. R.. (2011). Behavioral interpretations of intrinsic connectivity networks. J. Cogn. Neurosci. 23, 4022–4037. 10.1162/jocn_a_00077 21671731 PMC3690655

[B49] Latora V. Marchiori M. (2001). Efficient behavior of small-world networks. Phys. Rev. Lett. 87, 198701. 10.1103/PhysRevLett.87.198701 11690461

[B50] Lee J. M. Kyeong S. Kim E. Cheon K. A. (2016). Abnormalities of inter- and intra-hemispheric functional connectivity in autism spectrum disorders: a study using the autism brain imaging data exchange database. Front. Neurosci. 10, 191. 10.3389/fnins.2016.00191 27199653 PMC4853413

[B51] Lee M. H. Smyser C. D. Shimony J. S. (2013). Resting-state fMRI: a review of methods and clinical applications. Am. J. Neuroradiol. 34, 1866–1872. 10.3174/ajnr.A3263 22936095 PMC4035703

[B52] Lindquist M. A. Geuter S. Wager T. D. Caffo B. S. (2019). Modular preprocessing pipelines can reintroduce artifacts into fmri data Hum. Brain Map. 40, 2358–2376. 10.1002/hbm.24528 30666750 PMC6865661

[B53] Liu Z. Zhang Y. Yan H. (2012). Altered topological patterns of brain networks in mild cognitive impairment and Alzheimer's disease: a resting-state fMRI study. Psychiatr. Res. 202, 118–125. 10.1016/j.pscychresns.2012.03.002 22695315

[B54] Lord C. Rutter M. Couteur A. L. (1994). Autism diagnostic interview-revised: a revised version of a diagnostic interview for caregivers of individuals with possible pervasive developmental disorders. J. Autism Dev. Disord. 24, 659–685. 10.1007/BF02172145 7814313

[B55] Maximo J. O. Keown C. L. Nair A. Müller R. A. (2013). Approaches to local connec-tivity in autism using resting state functional connectivity MRI. Front. Hum. Neurosci. 7, 605. 10.3389/fnhum.2013.00605 24155702 PMC3792552

[B56] Minati L. Nigri A. Cercignani M. (2013). Detection of scale-freeness in brain connectivity by functional MRI: signal processing aspects and implementation of an open hardware co-processor. Med. Eng. Phys. 35, 1525–1531. 10.1016/j.medengphy.2013.04.013 23742932

[B57] Monk C. S. Peltier S. J. Wiggins J. L. Weng S. J. Carrasco M. Risi S.. (2009). Abnormalities of intrinsic functional connectivity in autism spectrum disorders. Neuroimage 47, 764–772. 10.1016/j.neuroimage.2009.04.069 19409498 PMC2731579

[B58] Mueller S. Wang D. Fox M. D. Yeo B. T. T. Sepulcre J. Sabuncu M. R.. (2013). Individual variability in functional connectivity architecture of the human brain. Neuron 77, 586–595. 10.1016/j.neuron.2012.12.028 23395382 PMC3746075

[B59] Nebel M. B. Joel S. E. Muscheli J. Barber A. D. Caffo B. S. Pekar J. J.. (2014). Disruption of functional organization within the primary motor cortex in children with autism. Hum. Brain Map. 35, 567–580. 10.1002/hbm.22188 23118015 PMC3864146

[B60] Nomi J. S. Uddin L. Q. (2015). Developmental changes in large-scale network connectivity in autism. Neuroimage Clin. 7, 732–741. 10.1016/j.nicl.2015.02.024 25844325 PMC4375789

[B61] Olivito G. Clausi S. Laghi F. Tedesco A. M. Baiocco R. Mastropasqua C.. (2016). Resting-state functional connectivity changes between dentate nucleus and cortical social brain regions in autism spectrum disorders. Cerebellum. 16, 283–297. 10.1007/s12311-016-0795-8 27250977

[B62] Padmanabhan A. Menon V. Lynch C. Schaer M. (2017). The default mode network in autism. Biol. Psychiatr. 2, 476–486. 10.1016/j.bpsc.2017.04.004 29034353 PMC5635856

[B63] Raff M. (2014). Open questions: what has genetics told us about autism spectrum disorders? BMC Biol. 12, 45. 10.1186/1741-7007-12-45 24903674 PMC4046436

[B64] Raichle M. E. Synder A. Z. (2007). A default mode of brain function: a brief history of an evolving idea. Neuroimage 37, 1083–1090. 10.1016/j.neuroimage.2007.02.041 17719799

[B65] Ray S. Miller M. Karalunas S. Robertson C. Grayson D. S. Cary R. P.. (2014). Structural and functional connectivity of the human brain in autism spectrum disorders and attention-deficit/hyperactivity disorder: a rich club- organization study. Hum. Brain Mapp. 35, 6032–6048. 10.1002/hbm.22603 25116862 PMC4319550

[B66] Redcay E. Moran J. M. Mavros P. L. Tager-Flusberg H. Gabrieli J. D. E. Whitfield-Gabrieli S.. (2013). Intrinsic functional network organization in high- functioning adolescents with autism spectrum disorder. Front. Hum. Neurosci. 7, 573. 10.3389/fnhum.2013.00573 24062673 PMC3777537

[B67] Richiardi J. Altmann A. Milazzo A. C. Chang C. Chakravarty M. M. Banaschewski T.. (2015). Correlated gene expression supports synchronous activity in brain networks. Science 2015, 1260670. 10.1126/science.1255905 26068849 PMC4829082

[B68] Ropar D. Mitchell P. (1999). Are individuals with autism and Asperger's syndrome susceptible to visual illusions? J. Child Psychol. Psychiatr. 40, 1283–1293. 10.1111/1469-7610.00544 10604406

[B69] Rubinove M. Sporns O. (2010). Complex network measures of brain connectivity: uses and interpretations. NeuroImage 52, 1059–1069. 10.1016/j.neuroimage.2009.10.003 19819337

[B70] Rudie J. D. Beck-Pancer D. Hernandez L. M. Dennis E. L. Thompson P. M. Bookheimer S. Y.. (2013). Altered functional and structural brain network organization in autism. Neuroimage Clin. 79–94. 10.1016/j.nicl.2012.11.006 24179761 PMC3777708

[B71] Rudie J. D. Dapretto M. (2013). Convergent evidence of brain overconnectivity in children with autism? Cell Rep. 5, 565–566. 10.1016/j.celrep.2013.10.043 24238089 PMC6396279

[B72] Ruggeri B. Sarkans U. Schumann G. Persico A. M. (2014). Biomarkers in autism spectrum disorder: the old and the new. Psychopharmacology 231, 1201–1216. 10.1007/s00213-013-3290-7 24096533

[B73] Sadeghi M. Khosrowabadi R. Bakouie F. Mahdavi H. Eslahchi C. Pouretemad H. (2017). Screening of autism based on task-free fMRI using gragh theoretical approach. Psychiatr. Res. 251, 1–7. 10.1016/j.pscychresns.2017.02.004 28324694

[B74] Smith S. M. Beckmann C. F. Ramnani N. Woolrich M. W. Bannister P. R. Jenkinson M.. (2005). Variability in fMRI: a re-examination of inter-session differences. Hum. Brain Mapp. 24, 248–257. 10.1002/hbm.20080 15654698 PMC6871748

[B75] Smith S. M. Fox P. T. Miller K. L. (2009). Correspondence of the brain's functional architecture during activation and rest. Proc. Natl. Acad. Sci. U. S. A. 106, 13040–13045. 10.1073/pnas.0905267106 19620724 PMC2722273

[B76] Smith S. M. Vidaurre D. Beckmann C. F. (2013). Functional connectomics from resting-state fMRI. Trends Cogn. Sci. 17, 666–682. 10.1016/j.tics.2013.09.016 24238796 PMC4004765

[B77] Sporns O. (2011). Networks of the Brain. Cambridge, MA: MIT Press. 10.7551/mitpress/8476.001.0001

[B78] Sporns O. (2013). Structure and function of complex brain networks. Dialog. Clin. Neurosci. 15, 247–262. 10.31887/DCNS.2013.15.3/osporns 24174898 PMC3811098

[B79] Sporns O. (2014). Contributions and challenges for network models in cognitive neuroscience. Nat. Neurosci. 17, 652–660. 10.1038/nn.3690 24686784

[B80] Sporns O. Tononi G. Kötter R. (2005). The human connectome: a structural description of the human brain. PLoS Comput. Biol. 1, e42. 10.1371/journal.pcbi.0010042 16201007 PMC1239902

[B81] Starck T. Nikkinen J. Rahko J. Remes J. Hurtig T. Haapsamo H.. (2013). Resting state fMRI reveals a default mode dissociation between retrosplenial and medial prefrontal subnetworks in ASD despite motion scrubbing. Front. Hum. Neurosci. 7, 802. 10.3389/fnhum.2013.00802 24319422 PMC3837226

[B82] Storey J. D. Tibshirani R. (2003). Statistical significance for genomewide studies. Proc. Natl. Acad. Sci. U. S. A. 100, 9440–9445. 10.1073/pnas.1530509100 12883005 PMC170937

[B83] Supekar K. Menon V. Rubin D. Musen M. Greicius M. D. (2008). Network analysis of intrinsic functional brain connectivity in Alzheimer's disease. PLoS Computat. Biol. 4, e1000100. 10.1371/journal.pcbi.1000100 18584043 PMC2435273

[B84] Supekar K. Uddin L. Q. Prater K. Amin H. Greicius M. D. Menon V.. (2010). Development of functional and structural connectivity within the default mode network in young children. Neuroimage 52, 290–301. 10.1016/j.neuroimage.2010.04.009 20385244 PMC2976600

[B85] Thomason M. E. Dennis E. L. Joshi A. A. Joshi S. H. Dinov I. D. Chang C.. (2011). Resting-state fMRI can reliably map neural networks in children. Neuroimage 55, 2157–2172. 10.1016/j.neuroimage.2010.11.080 21134471 PMC3031732

[B86] Tyszka J. M. Kennedy D. P. Paul L. K. Adolphs R. (2014). Largely typical patterns of resting-state functional connectivity in high-functioning adults with autism. Cereb. Cortex 24, 1894–1905. 10.1093/cercor/bht040 23425893 PMC4051895

[B87] Uddin L. Q. Supekar K. Lynch C. J. Cheng K. M. Odriozola P. Barth M. E.. (2015). Brain state differentiation and behavioral inflexibility in autism. Cereb. Cortex 25, 4740–4747. 10.1093/cercor/bhu161 25073720 PMC4635916

[B88] van den Heuvel M. P. Hulshoff Pol H. E. (2010). Exploring the brain network: a review on resting-state fMRI functional connectivity. Eur. Neuropsychopharmacol. 20, 519–534. 10.1016/j.euroneuro.2010.03.008 20471808

[B89] van den Heuvel M. P. Stam C. J. Boersma M. (2008). Small-world and scale-free organization of voxel-based resting-state functional connectivity in the human brain. Neuroimage 43, 528–539. 10.1016/j.neuroimage.2008.08.010 18786642

[B90] Verly M. Verhoeven J. Zink I. Mantini D. Peeters R. Deprez S.. (2014). Altered functional connectivity of the language network in ASD: role of classical lan-guage areas and cerebellum. Neuroimage Clin. 4, 374–382. 10.1016/j.nicl.2014.01.008 24567909 PMC3930113

[B91] von dem Hagen E. A. Stoyanova R. S. Baron-Cohen S. Calder A. J. (2013). Reduced functional connectivity within and between ‘social' resting state networks in autism spectrum conditions. Soc. Cogn. Affect. Neurosci. 8, 694–701. 10.1093/scan/nss053 22563003 PMC3739917

[B92] Wang D. Buckner R. L. Liu H. (2014). Functional specialization in the human brain estimated by intrinsic hemispheric interaction. J. Neurosci. 34, 112341–112352. 10.1523/JNEUROSCI.0787-14.2014 25209275 PMC4160771

[B93] Wang J. Wang L. Zang Y. (2009). Parcellation-dependent small-world brain functional networks: a resting-state fMRI study. Hum. Brain Mapp. 30, 1511–1523. 10.1002/hbm.20623 18649353 PMC6870680

[B94] Wang J. Zuo X. He Y. (2010). Graph-based network analysis of resting-state functional MRI. Front. Syst. Neurosci. 4, 16. 10.3389/fnsys.2010.00016 20589099 PMC2893007

[B95] Washington S. D. Gordon E. M. Brar J. Warburton S. Sawyer A. T. Wolfe A.. (2014). Dysmaturation of the default mode network in autism. Hum. Brain Mapp. 35, 1284–1296. 10.1002/hbm.22252 23334984 PMC3651798

[B96] Weng S. J. Wiggins J. L. Peltier S. J. Carrasco M. Risi S. Lord C.. (2010). Alterations of resting state functional connectivity in the default network in adolescents with autism spectrum disorders. Brain Res. 1313, 202–214. 10.1016/j.brainres.2009.11.057 20004180 PMC2818723

[B97] West P. R. Amaral D. G. Bais P. Smith A. M. Egnash L. A. Ross M. E.. (2014). Metabolomics as a tool for discovery of biomarkers of autism spectrum disorder in the blood plasma of children. PLoS ONE 9, e112445. 10.1371/journal.pone.0112445 25380056 PMC4224480

[B98] Wiggins J. L. Peltier S. J. Ashinoff S. Weng S. J. Carrasco M. Welsh R. C.. (2011). Using a self-organizing map algorithm to detect age-related changes in functional connectivity during rest in autism spectrum disorders. Brain Res. 1380, 187–197. 10.1016/j.brainres.2010.10.102 21047495 PMC3050117

[B99] Yeo B. T. Krienen F. M. EicKhoff S. B. Yaakub S. N. Fox P. T. Buckner R. L.. (2014). Functional Specialization and Flexibility in Human Association Cortex. Oxford: Oxford University Press. 10.1093/cercor/bhu217

[B100] Yeo B. T. Krienen F. M. Sepulcre J. Sabuncu M. R. Lashkari D. Hollinshead M.. (2011). The organization of the human cerebral cortex estimated by intrinsic functional connectivity. J. Neurophysiol. 106, 1125–1165. 10.1152/jn.00338.2011 21653723 PMC3174820

[B101] Yerys B. E. Gordon E. M. Abrams D. N. Satterthwaite T. D. Weinblatt R. Jankowski K. F.. (2015). Default mode network segregation and social deficits in autism spectrum disorder: evidence from non-medicated children DMN in chil-dren with ASD. Neuroimage Clin. 9, 223–232. 10.1016/j.nicl.2015.07.018 26484047 PMC4573091

[B102] You X. Norr M. Murphy E. Kuschner E. S. Bal E. Gaillard W. D.. (2013). Atypical modulation of distant functional connectivity by cognitive state in children with autism spectrum disorders. Front. Hum. Neurosci. 7, 482. 10.3389/fnhum.2013.00482 23986678 PMC3753572

[B103] Zuo X. N. Kelly C. Adelstein J. S. Klein D. F. Castellanos F. X. Milham M. P.. (2010). Reliable intrinsic connectivity networks: test-retest evaluation using ICA and dual regression approach. Neuroimage 49, 2163–2177. 10.1016/j.neuroimage.2009.10.080 19896537 PMC2877508

