# Supplementary material for: Cognitive theories of autism based on the interactions between brain functional networks
Source: Front Hum Neurosci. 2022 Oct 6;16:828985. doi: 10.3389/fnhum.2022.828985 (PMC9614840; doi:10.3389/fnhum.2022.828985)
Supplement: Supplementary file 1 [file Data_Sheet_1.docx]

**Supplementary materials**

Table 1 presents the pairs of networks which are significantly different in ASDs as compared to HCs, according to the parcellation used by Yeo et al. (p<0.05, FDR corrected). Table 2 presents the pairs of networks which are significantly different in ASDs as compared to HCs, according to the parcellation used by Thomason et al. (17 networks) (p<0.05, FDR corrected).

**Table 1.** Significant changes of interactions between brain functional networks in ASD as compared to HC based on Yeo et al. parcellation method

| **Networks** | **ASD**  (mean ± sd) | **HC**  (mean ± sd) | **Effect size** | **p-value** | **t-value** | **Observed Power** |
| --- | --- | --- | --- | --- | --- | --- |
| Visual-A and DMN-C | 0.2752±0.2068 | 0.1954±0.2282 | 0.388 | 0.042 | 2.062 | 0.533 |
| Visual-B and DMN-C | 0.2986±0.2076 | 0.2143±0.2388 | 0.405 | 0.018* | 2.401 | 0.662 |
| Visual-B and Fronto-parietal-A | 0.5783±0.1382 | 0.5026±0.1724 | 0.550 | 0.003* | 2.437 | 0.855 |
| Ventral-attention-B and DMN-C | 0.3828±0.2457 | 0.3518±0.2570 | 0.126 | 0.050 | 1.980 | 0.500 |

Abbreviations: sd – standard deviation; * denotes P value <0.05, FDR corrected.

**Table** **2.** Significant changes of interactions between brain functional networks in ASD as compared to HC based on Thomason et al. parcellation method

| **Networks** | **ASD**  (mean ± sd) | **HC**  (mean ± sd) | **Effect size** | **p-value** | **t-value** | **Observed Power** |
| --- | --- | --- | --- | --- | --- | --- |
| Posterior default and Visual | 0.7199±0.0924 | 0.6494±0.1468 | 0.760 | 0.002* | 3.171 | 0.881 |
| Posterior default and IFG-middle-Temporal | 0.8877±0.5508 | 0.8749±0.5639 | 0.023 | 0.050 | 1.983 | 0.501 |

Abbreviations: sd – standard deviation; * denotes P value <0.05, FDR corrected.

**Table 3.** Significant decreases in betweenness centrality of brain networks in ASD as compared to HC based on Yeo et al. and Thomason et al. parcellation methods

| **Networks** | **ASD**  (mean ± sd) | **HC**  (mean ± sd) | **Effect size** | **p-value** | **t-value** | **Observed Power** |
| --- | --- | --- | --- | --- | --- | --- |
| Somatomotor  (Yeo et al, 2011) | 1.040±1.873 | 2.470±3.520 | -0.763 | 0.018 * | 2.405 | 0.663 |
| Dorsal-attention-B (Yeo et al, 2011) | 5.000±6.663 | 6.900±7.657 | -0.285 | 0.048 | 2.002 | 0.509 |
| Limbic-A  (Yeo et al, 2011) | 2.090±4.194 | 3.570±6.261 | -0.352 | 0.045 | 2.027 | 0.519 |
| IFG-middle-temporal (Thomason et al., 2010) | 1.435±2.721 | 2.900±4.205 | -0.538 | 0.036 | 2.12 | 0.556 |

Abbreviations: sd – standard deviation; * denotes P value <0.05, FDR corrected.
